# Pulmonary endarterectomy for chronic thromboembolic pulmonary hypertension after bilateral lung transplantation

**DOI:** 10.1016/j.jhlto.2025.100385

**Published:** 2025-09-09

**Authors:** Bianca Battilana, Jan Mengers, György Lang, Martina Haberecker, Claudio Caviezel, Macé M. Schuurmans, Silvia Ulrich, Isabelle Opitz

**Affiliations:** a University Hospital Zurich, Department of Thoracic Surgery, Zurich, Switzerland; b University Hospital Zurich, Department of Pathology, Zurich, Switzerland; c University Hospital Zurich, Department of Pulmonology, Zurich, Switzerland; d University of Zurich, Zurich, Switzerland

**Keywords:** Pulmonary endarterectomy, Lung transplantation, Chronic Thromboembolic Pulmonary Hypertension

## Abstract

A 30-year-old woman suffering from diffuse interstitial lung disease underwent bilateral lung transplantation in 2008. A high-risk bilateral pulmonary embolism (PE) occurred in 2020 after deep venous thrombosis. After lysis (Endovascular System and oral anticoagulation), 1-year follow-up imaging showed a persisting thrombus-load (Fig. 1). Right heart catheterization (RHC) 18 months after PE showed s/m/dPAP of 51/34/25mmHg, PVR of 5.6WU and CO 4.43l/min. Echocardiography demonstrated right ventricular dilation with impaired radial and longitudinal function. She reported worsening dyspnea (NYHA III) and impaired lung function (FEV1 1.3 l, DLCO 43%, best post-transplant value: FEV1 2.44 l, DLCO 68%) without diagnosis of CLAD. CT-imaging showed organized thrombotic material in the right inferior lobe and left pulmonary artery (Fig. 1). In 2022, bilateral PEA under deep hypothermic circulatory arrest was performed. On the right, multiple segments were obliterated, whereas in the left main trunk a cone-shaped occlusion of the complete left main PA at the level of the anastomosis was resected in addition to peripheral lesions (Fig. 1 and 2). Histopathological evaluation revealed cartilaginous metaplasia of the tunica intima (Fig. 2). This phenomenon represents a vascular remodeling process in which chronic thromboembolic injury, inflammation, TGF-*β*1-mediated vascular smooth muscle cell transdifferentiation, and transplant-related stress collectively drive the formation of cartilage-like tissue, resulting in irreversible maladaptive remodeling and pulmonary hypertension.

The patient was extubated on the first postoperative day and discharged to rehabilitation after 14 days. After 7 weeks, she reported improved performance (NYHA I), and echocardiography showed decreased sPAP (from 45 to 37 mmHg). 18-month postoperatively the patient was in an excellent condition, reported further improvements in performance (NYHA I) and RHC showing s/m/dPAP of 30/19/11mmHg, PVR 4.1WU and CO 3.65l/min.

## Introduction

Chronic thromboembolic pulmonary hypertension (CTEPH) is a rare and debilitating disease characterized by unresolved thromboembolisms leading to bilateral pulmonary arterial stenosis, increased pulmonary vascular resistance (PVR), pulmonary hypertension (mPAP≥20mmHg) and progressive right heart failure.^1^ Pulmonary endarterectomy (PEA) is the treatment of choice for patients with surgically accessible lesions.^2^

PEA after organ transplantation is extremely rare. To date, only three cases of successful PEA for CTEPH in heart transplant recipients were reported.^3^, ^4^ PEA after lung transplantation has never been described. We report the first documented case of PEA performed after bilateral lung transplantation.

### Case Report

**Figure 1:**
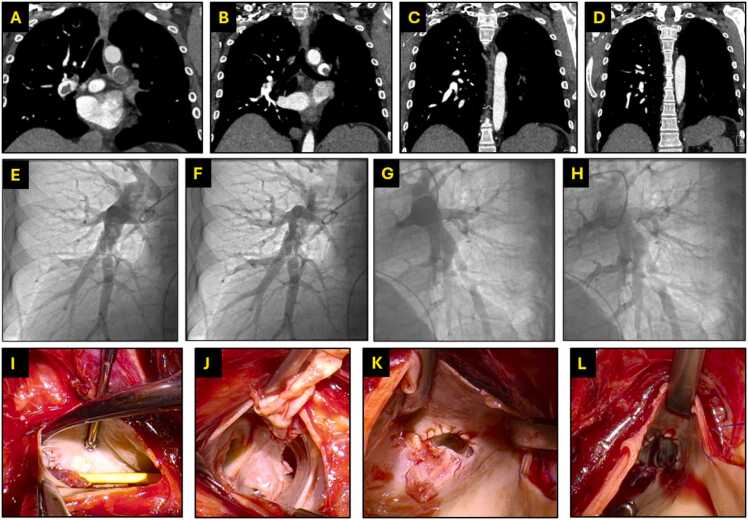
Preoperative CT-scans and intraoperative situs. A: acute thrombotic material in the right inferior lobe artery and in the left pulmonary artery with complete occlusion at the time point of acute PE. B: Follow-up imaging after 18 months shows organized thrombotic material in the right inferior lobe artery and in the left pulmonary artery. C & D: Preoperative CT-scan showing peripheral webs and bands on the right side and almost completely obliterated arteries on the left side. E & F: Preoperative pulmonary angiography of the right side. G & H: Preoperative pulmonary angiography of the left side. I & J: Chronic thromboembolic material in the right pulmonary artery during PEA. K & L: Thrombotic material in the left pulmonary artery appearing as a blind bag at the level of the former anastomosis. D: Desobliterated left pulmonary artery with unimpaired former anastomosis.

**Figure 2:**
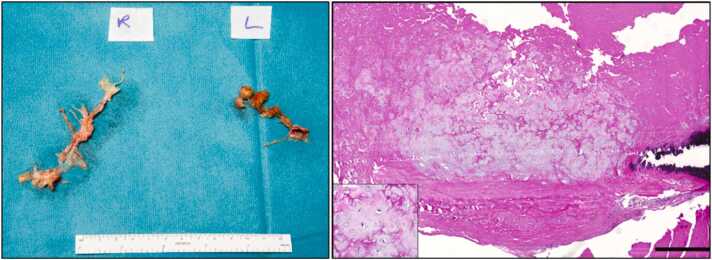
Left: Specimen of endarterectomized material from the pulmonary arteries. Right: Tunica intima resection with centrally located cartilaginous metaplasia. On the right-side additional calcification. Insert: Chondrocytes embedded in cartilage matrix. Scale bar 0.5 mm.

A 30-year-old woman suffering from diffuse interstitial lung disease underwent bilateral lung transplantation in 2008. A high-risk bilateral pulmonary embolism (PE) occurred in 2020 after deep venous thrombosis. After lysis (Endovascular System and oral anticoagulation), 1-year follow-up imaging showed a persisting thrombus-load (Figure 1). Right heart catheterization (RHC) 18 months after PE showed s/m/dPAP of 51/34/25mmHg, PVR of 5.6WU and CO 4.43l/min. Echocardiography demonstrated right ventricular dilation with impaired radial and longitudinal function. She reported worsening dyspnea (NYHA III) and impaired lung function (FEV1 1.3 l, DLCO 43%, best post-transplant value: FEV1 2.44 l, DLCO 68%) without diagnosis of CLAD. CT-imaging showed organized thrombotic material in the right inferior lobe and left pulmonary artery (Fig. 1). In 2022, bilateral PEA under deep hypothermic circulatory arrest was performed. On the right, multiple segments were obliterated, whereas in the left main trunk a cone-shaped occlusion of the complete left main PA at the level of the anastomosis was resected in addition to peripheral lesions (Figure 1, Figure 2). Histopathological evaluation revealed cartilaginous metaplasia of the tunica intima (Figure 2). This phenomenon represents a vascular remodeling process in which chronic thromboembolic injury, inflammation, TGF-β1–mediated vascular smooth muscle cell transdifferentiation, and transplant-related stress collectively drive the formation of cartilage-like tissue, resulting in irreversible maladaptive remodeling and pulmonary hypertension.^5^

The patient was extubated on the first postoperative day and discharged to rehabilitation after 14 days. After 7 weeks, she reported improved performance (NYHA I), and echocardiography showed decreased sPAP (from 45 to 37 mmHg). 18-month postoperatively the patient was in an excellent condition, reported further improvements in performance (NYHA I) and RHC showing s/m/dPAP of 30/19/11mmHg, PVR 4.1WU and CO 3.65l/min.

## Discussion

PEA is a highly selective intervention but stands out as a viable option for patients with CTEPH post-transplantation. PEA after a heart transplantation can be performed by specialized teams at high-volume transplantation centers and lead to positive outcomes for patients.^3^, ^4^ We illustrate that PEA is feasible for patients with CTEPH following bilateral lung transplantation despite increased risk of previous surgery and anastomoses between recipient and donor. PEA offered substantial benefits such as significant improvements in this young patients' performance, hemodynamic parameters, and quality of life.

Our case contributes to the scarce evidence supporting feasibility and effectiveness of PEA in post-transplant settings in well-selected patients. It is crucial to consider these positive outcomes when evaluating the risk of treatment options for CTEPH patients after organ transplantation, as they highlight the potential for improved quality of life and clinical prognosis.

## Informed consent

Written informed consent was obtained from the patient for publication of this case report and accompanying images. A copy of the written consent is available for review by the Editor-in-Chief of this journal upon request.

## Funding statement

No funding was received for this work.

## Disclosure statement

IO reports the following disclosures: Roche (Institutional Grant and Speakers Fee), Roche Genentech (Steering Committee), AstraZeneca (Advisory Board and Speakers Fee), MSD (Advisory Board), BMS (Advisory Board), Medtronic (Institutional Grant and Advisory Board), Intuitive (Proctorship and Speakers Fee), Sanofi (Speakers Fee), Regeneron (Advisory Board), XVIVO (Institutional Grant), Siemens (Speaker). JM, HM, MMS, GL, CC, SU and BB have no disclosures and no conflict of interest.

## Declaration of Competing Interest

The authors declare the following financial interests/personal relationships which may be considered as potential competing interests: IO has no real conflicts of interest. The following could be perceived as such: Roche (Institutional Grant), AstraZeneca (Advisory Board and Steering Committee), MSD (Advisory Board), BMS (Advisory Board), Medtronic (Institutional Grant and Advisory Board), Intuitive (Proctorship and Speakers Fee), Sanofi (Speakers Fee), Regeneron (Advisory Board), XVIVO (Institutional Grant), Siemens (Speakers Fee), Astellas (Speakers Fee). IO is IASLC Board Director, Member of the Thoracic Clinical Practice Standards Committee and the Thoracic Education Committee of AATS, iMig Board Member and JTCVS Associate Editor. She is in the Stiftungsrat Schulthessklinik and an Advisory Board Member at Med Uni Wien for Comprehensive Center for Chest Diseases (CCCD). The other authors declare that they have no known competing financial interests or personal relationships that could have appeared to influence the work reported in this paper.

## References

[1] Humbert M., Kovacs G., Hoeper M.M. (Oct 11 2022). 2022 ESC/ERS Guidelines for the diagnosis and treatment of pulmonary hypertension. Eur Heart J.

[2] Jenkins D., Madani M., Fadel E., D'Armini A.M., Mayer E. (Jan 2017). Pulmonary endarterectomy in the management of chronic thromboembolic pulmonary hypertension. Eur Respir Rev.

[3] Moiroux-Sahraoui A., Issard J., Menager J.B. (Nov 2023). Successful pulmonary endarterectomy after heart transplantation. J Heart Lung Transplant.

[4] Bigdeli A.K., Beiras-Fernandez A., Kaczmarek I. (Jun 2007). Successful pulmonary thromboendarterectomy for right atrial thrombosis in a heart transplant recipient: a case report. Exp Clin Transplant.

[5] Mathieu P., Roussel J.C., Dagenais F., Anegon I. (Nov 2003). Cartilaginous metaplasia and calcification in aortic allograft is associated with transforming growth factor beta 1 expression. J Thorac Cardiovasc Surg.

